# Value of the cell cycle arrest biomarkers in the diagnosis of pregnancy-related acute kidney injury

**DOI:** 10.1042/BSR20200962

**Published:** 2021-01-05

**Authors:** Osama El Minshawy, Mahmoud Hassan Sayed Khedr, Ayman Moheb Youssuf, Mostafa Abo Elela, Fatma Mohamed Mohamed Kamel, Hesham Kamal Habeeb Keryakos

**Affiliations:** 1 Department of Internal Medicine, Minia Faculty of Medicine, Minia University Hospital, Minia, Egypt; 2 Department of Obstetrics and Gynecology, Minia Faculty of Medicine, Minia University Hospital, Minia, Egypt; 3 Department of Clinical Pathology, Minia Faculty of Medicine, Minia University Hospital, Minia, Egypt

**Keywords:** Cell-cycle arrest biomarkers, Insulin-like growth factor-binding protein 7 (IGFBP7), Pregnancy related acute kidney injury, Tissue inhibitor of metalloproteinases-2 (TIMP-2)

## Abstract

Background: Pregnancy-related acute kidney injury (PRAKI) is still a common serious problem in developing countries. Insulin-like growth factor-binding protein 7 (IGFBP7) and tissue inhibitor metalloproteinases-2 (TIMP-2) can identify critically ill patients at risk for the development of severe AKI. Aim: To identify main causes and timing of PRAKI and to study the G1 cell cycle arrest biomarkers in cases diagnosed with (PRAKI) as a diagnostic tool. Methods: 80 pregnant women diagnosed with PRAKI were recruited from a single hospital as well as 30 age-matched pregnant women with normal pregnancy participated in the present study. A urine specimen was collected from all study participants with established AKI within 24 h of ICU admission to measure [TIMP-2]*[IGFBP7]. Results: The incidence of PRAKI was 1.1%. The most common cause of PRAKI is pre-eclampsia/eclampsia spectrum (61%). Most of the cases occur in the third trimester (60%) and postpartum period (23%). At a cutoff 0.33 ng/ml, the estimated sensitivity and specificity of urinary [TIMP-2]*[IGFBP7] in predicting PRAKI is 100% (95% CI) with NPV and PPV are 100%. Conclusion: Urinary [TIMP-2]*[IGFBP7] serves as a sensitive and specific biomarker in the diagnosis of PRAKI.

## Introduction

Pregnancy-related acute kidney injury (PRAKI) is still a common issue in developing countries with serious consequences on the mother and fetus [1]. Consensus definitions of AKI used in the general population can mask AKI in early disease course as glomerular filtration rate (GFR) increases significantly during pregnancy resulting in lower serum creatinine as compared with healthy nonpregnant women, so they have not been validated in pregnancy.

Acute kidney injury in pregnancy is defined as a serum creatinine level of >1.1 mg/dl or a doubling of the serum creatinine concentration in the absence of other renal disease [2]. Formulas used to estimate GFR based on creatinine in nonpregnant women are not reliable in pregnant women with only timed urine creatinine excretion is helpful [3].

PRAKI occurs mainly during the third trimester and postpartum period. The etiology of PRAKI is highly variable depending on the country and trimester of pregnancy. Hypertensive complications of pregnancy, namely pre-eclampsia and eclampsia, are the leading cause of PRAKI in both developed and developing countries. Pregnancy and its complications play a predominant primary role in the genesis of PRAKI in most cases, or in few cases act as a trigger in genetically susceptible patients [4].

The biomarker [TIMP-2]*[IGFBP7] panel has been shown to predict future development of AKI within the next 12 h [5]. Then, these two biomarkers was validated in a second study (Sapphire) with 744 adult patients with critical illness and without evidence of AKI at enrollment with a primary end point of AKI defined as KDIGO stage 2 or 3, which was developed in 14% of participants and the biomarkers performed moderately well in the prediction of AKI [6]. Then, the utility of urine [TIMP-2]*[IGFBP7] has been investigated in different patient populations including infants (<1 year of age) undergoing cardiac surgery with cardiopulmonary bypass [5], adults following cardiac surgery [7–9], and exposures to nephrotoxins/renal insults [10]. Patients with elevated urinary [TIMP-2]*[IGFBP7] showed improved AKI outcomes in with the provision of AKI-focused care in several RCTs [11–13]. In the present study, we identify the main causes and timing of PRAKI and to study the G1 cell cycle arrest biomarkers in cases diagnosed with (PRAKI) as a diagnostic tool and if they have a role in differentiating the different causes of PRAKI.

## Subjects and methods

### Study participants

The study was reviewed and approved by the local institutional ethics and review committee of Minia University Hospital (MU1562017) and was conducted in accordance with the Helsinki Declaration. Informed consent was obtained from all participants. The present study was an observational study to study the cause of PRAKI admitted to the hospital and to assess the sensitivity and specificity of cell cycle arrest urinary biomarkers; tissue inhibitor of metalloproteinases-2 (TIMP-2), and insulin-like growth factor-binding protein 7 (IGFBP7) in the diagnosis of PRAKI, with the participants recruited from the emergency room and critical care unit of Minia University Hospital over the period from June 2017 to December 2018. The study enrolled 80 pregnant patients with PRAKI out of 7253 deliveries during this period; as well as 30 age- and sex-matched healthy pregnant controls. The inclusion criteria were all pregnant and postpartum patients who develop PRAKI. Exclusion criteria were pregnant patients with pre-existing renal disease. A urine specimen was collected from each participant within 24 h of admission to ICU and kept frozen at −80° until further analysis for IGFBP7 and TIMP-2 then the results multiplied ([TIMP-2]*[IGFBP7]) expressed in (ng/ml) 2/1000.

The diagnosis of AKI in pregnancy was based on the following criteria (any one of three): Serum creatinine > 1.1 mg/dl, doubling of serum creatinine from baseline, oliguria/anuria > 12 h duration, or the need for dialysis [2]. Then patients with AKI were divided into three categories based on constellation of symptoms and signs, laboratory findings including fractional excretion of sodium (FeNa) and imaging. Pre-eclampsia is defined as blood pressure ≥140/90 mmHg on two occasions at least 4 h apart or ≥160/110 mmHg within a shorter interval (minutes), at ≥20 weeks of gestation, in women with previously normal blood pressure and proteinuria. Proteinuria is defined as urinary protein excretion ≥300 mg/24 h, a total protein:creatinine ratio ≥30 mg/mmol (or ≥0.3 when both are measured in mg/dl) or a dipstick reading of ≥1+ (only if other quantitative methods are not available). In the absence of proteinuria, new-onset hypertension plus new onset of any of the following features: serum creatinine concentrations >1.1 mg/dl or doubling of serum creatinine concentration in the absence of other renal disease; elevation of liver transaminases to twice normal concentration; pulmonary edema; and new-onset cerebral or visual disturbances. Eclampsia is defined as seizures in women with pre-eclampsia that cannot be attributed to other causes [14].

### Human insulin like growth factor binding protein 7 (IGFBP7)

Urine samples were drawn within 24 h of ICU admission from each of the study participants with established AKI. Measurements of urine IGFBP7 concentrations were performed using a two-site second-generation Enzyme-Linked Immunosorbent Assay (ELISA) Kit (Bioassay Technology Laboratory, Shanghai Korain Biotech Co. Ltd., China) with reference range is 0.05–20 ng/ml, intra-assay precision is CV<8%, and inter-assay precision is CV<10%. The microtiter plate was coated with monoclonal anti-IGFBP7 antibody. About 50 μl of standards or samples are added to the appropriate microtiter plate wells, and incubate for 60 min at 37°C. Remove the liquid of each well; add 50 μl of a biotin-conjugated polyclonal anti-IGFBP7 antibody to each well and incubate for 1 h at 37°C. Aspirate each well and wash with wash buffer, repeating the process three times for a total of three washes, followed by the addition of 50 μl of Avidin conjugated to horseradish peroxidase (HRP) to each microplate well and incubated for 30 min at 37°C. aspirate and wash five times. Color development was achieved using a 50 μl TMB substrate solution is added to each well and incubated for 10 min at 37°C in the dark. Only those wells that contain, biotin-conjugated antibody and enzyme-conjugated Avidin will exhibit a change in color. The enzyme–substrate reaction is terminated by the addition of 50 μl sulphuric acid solution and the color change is measured spectrophotometrically at a wavelength of 450 nm ± 2 nm. Serial dilutions of recombinant human IGFBP7 were used to establish a standard curve.

### Human tissue inhibitors of metalloproteinase 2 (TIMP-2)

Urine samples were drawn within 24 h of ICU admission from each of the study participants with established AKI. Measurements of urine TIMP-2 concentrations were performed using a two-site second-generation Enzyme-Linked Immunosorbent Assay (ELISA) Kit (Bioassay Technology Laboratory, Shanghai Korain Biotech Co. Ltd., China) with reference range is 0.05–200 ng/ml, intra-assay precision is CV<8%, and inter-assay precision is CV<10%. The microtiter plate was coated with monoclonal anti-TIMP-2 antibody. About 50 μl of standards or samples are added to the appropriate microtiter plate wells, and incubate for 60 min at 37°C. Remove the liquid of each well; add 50 μl of a biotin-conjugated polyclonal anti-TIMP-2 antibody to each well and incubate for 1 h at 37°C. Aspirate each well and wash with wash buffer, repeating the process three times for a total of three washes, followed by the addition of 50 μl of Avidin conjugated to horseradish peroxidase (HRP) to each microplate well and incubated for 30 min at 37°C. aspirate and wash five times. Color development was achieved using a 50 μl TMB substrate solution is added to each well and incubated for 10 min at 37°C in the dark. Only those wells that contain, biotin-conjugated antibody and enzyme-conjugated Avidin will exhibit a change in color. The enzyme–substrate reaction is terminated by the addition of 50 μl sulphuric acid solution and the color change is measured spectrophotometrically at a wavelength of 450 nm ± 2 nm. Serial dilutions of recombinant human TIMP-2 were used to establish a standard curve.

### Other measurements

Demographic data with maternal history including gestational age, parity, gravidity, and maternal and fetal complications as well as physical examination were obtained at enrollment. Complete blood cell count (CBC), prothrombin time and concentration, INR, APTT, C-reactive protein (CRP), renal function tests, liver function tests were measured using stored ethylenediaminetetraacetic acid (EDTA) plasma samples using standard techniques. Complete urine analysis, spot urine protein/creatinine ratio and abdominal ultrasound were performed in all patients. Serological investigations such as ANA, anti-dsDNA, C3, C4, LDH, anticardiolipin antibodies, lupus anticoagulant assay and β2 glycoprotein were performed in selected patients. Kidney biopsy was performed only in patients with anuria and/or oligo-anuria of >4 weeks duration and in those with partial recovery of renal function after 3 months of the diagnosis of AKI.

### Statistical analysis

For the primary analysis, and to compare the difference between the study groups independent samples *T* test for parametric quantitative data between the two groups, Mann–Whitney test for nonparametric quantitative data (expressed as median) between the two groups and Fisher’s exact test for qualitative data between the two groups. For all tests *P*-values #0.05 were considered significant. To analyze the predictive power of these biomarkers receiver operating characteristic curves (ROC) were calculated and the area under the ROC curve (AUC) was determined. 95% confidence intervals (CI) were reported.

## Results

### Clinical and laboratory characteristics of the study participants

Table 1 shows both groups are age matched. It shows also significant increase in systolic and diastolic blood pressure in PRAKI groups in relation to control group. As regard complete blood count, patients in the PRAKI group showed statistically significant lower hemoglobin (8 ± 2 vs. 11 ± 1; *P****<***0.001), white blood cells count (14 ± 8 vs. 6 ± 1; *P***<**0.001), platelets count (169 ± 133 vs. 265 ± 45; *P*<0.001). With respect liver function tests, PRAKI showed statistically significant higher INR (1 ± 0.4 vs. 1 ± 0; *P*<0.001), AST (258.8 ± 1431.7 vs. 19 ± 6; *P*<0.001), ALT (140 ± 504.5 vs. 19 ± 7; *P*<0.001), total bilirubin (2 ± 4 vs. 0.8 ± 0.3; *P*<0.001), and direct bilirubin (1 ± 3 vs. 0.3 ± 0.1; *P*<0.001). Importantly, renal function showed statistically significant higher blood urea (110 ± 66 vs. 22 ± 3; *P*<0.001) and serum creatinine (1.6 ± 1.4 vs. 0.4 ± 0.1; *P*<0.001) in PRAKI group as compared with control group.

**Table 1:** Demographic, clinical characteristics among study groups

	Control	Cases	*P* value
	*N*=30	*N*=80	
**Age (years)**			
Range	(20–34)	(17–37)	0.646
Mean ± SD	25.6 ± 4.6	26.1 ± 4.9	
**Systolic BP (mmHg)**			
Range	(90–130)	(120–210)	<0.001*
Mean ± SD	111 ± 13	147 ± 27	
**Diastolic BP (mmHg)**			
Range	(60–80)	(67–160)	<0.001*
Mean ± SD	71 ± 10	92 ± 15	
**Diabetes mellitus**			
Yes	0	0	**1**
No	30	80	
**Hemoglobin (gm/dl)**			
Range	(10–12)	(5–13)	<0.001*
Mean ± SD	11 ± 1	8 ± 2	
**WBCs (×10^3^)**			
Range	(4–7)	(4–45)	<0.001*
Mean ± SD	6 ± 1		**14** ± **8**
Median	6	14	
**Platelets (×10^3^)**			
Range	(190–325)	(33–686)	<0.001*
Mean ± SD	265 ± 45	169 ± 133	
Median	270	116	
**INR**			
Range	(1–1)	(0.9–4)	<0.001*
Mean ± SD	1 ± 0	1.213 ± 0.4	
**AST (IU/l)**			
Range	(12–33)	(10–1056)	<0.001*
Mean ± SD	19 ± 6	258.8 ± 1431.7	
Median	18	28	
**ALT (IU/l)**			
Range	(10–33)	(10–704)	<0.001*
Mean ± SD	19 ± 7	140±504.5	
Median	19	25.5	
**Total bilirubin (mg/dl)**			
Range	(0.3–1.2)	(0.2–26)	<0.001*
Mean ± SD	0.8 ± 0.3	2 ± 4	
Median	0.9	1.1	
**Direct bilirubin (mg/dl)**			
Range	(0.1-0.5)	(0.1–16)	<0.001*
Mean ± SD	0.3±0.1	1 ± 3	
Median	0.3	0.6	
**Blood urea (mg/dl)**			
Range	(15–26)	(36–270)	<0.001*
Mean ± SD	22 ± 3	110 ± 66	
Median	22	99	
**Serum creatinine (mg/dl)**			
Range	(0.3–0.6)	(0.5–9)	<0.001*
Mean ± SD	0.4 ± 0.1	1.6 ± 1.4	
Median	0.5	3	

• ALT, alanine transaminase; AST, aspartate transaminase; Hb, hemoglobin; INR, international normalized ratio; WBC, white blood cell.

• Laboratory tests were done at the time of urine sampling.

### The most vulnerable period of PRAKI is the third trimester

**Figure 1:**
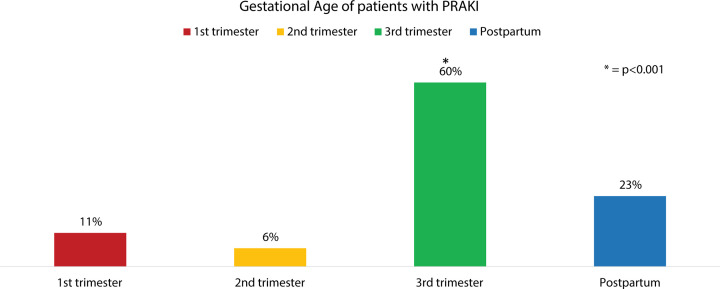
Gestational age of patients with PRAKI and their relative frequencies Most cases occur in the third trimester 60% as compared with first trimester (11%), second trimester (6%) or postpartum period (23%).

Most of the PRAKI occurs in the third trimester (48 patients = 60%) as compared with postpartum period (18 patients = 23%) followed by first trimester (9 patients = 11%), and finally second trimester (5 patients = 6%) (Figure 1).

### Obstetric complications that predispose to PRAKI

**Figure 2:**
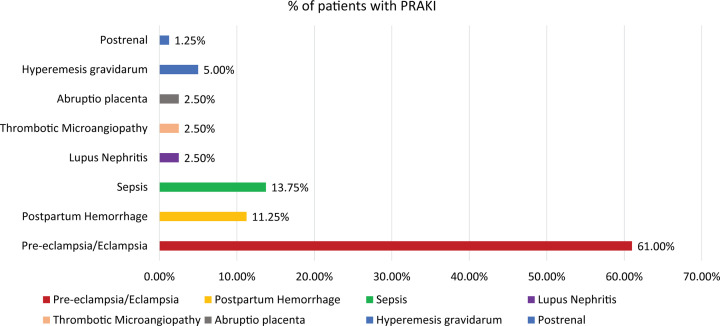
Relative frequencies of patients with obstetric complications that contribute to PRAKI

The most common obstetric complication that leads to PRAKI is hypertensive disorders of pregnancy, namely pre-eclampsia and eclampsia that contribute collectively to 61% of the cases. The second common cause was sepsis attributed to intrauterine fetal death (IUFD), premature rupture of membranes and postoperative sepsis. This is followed by postpartum hemorrhage with subsequent ischemic acute tubular necrosis. Other causes contribute to only 14% of cases which include hyperemesis gravidarum, abruptio placenta, thrombotic microangiopathy, lupus nephritis and postrenal obstruction (Figure 2).

### Intrinsic renal AKI is the most common cause of PRAKI

**Figure 3:**
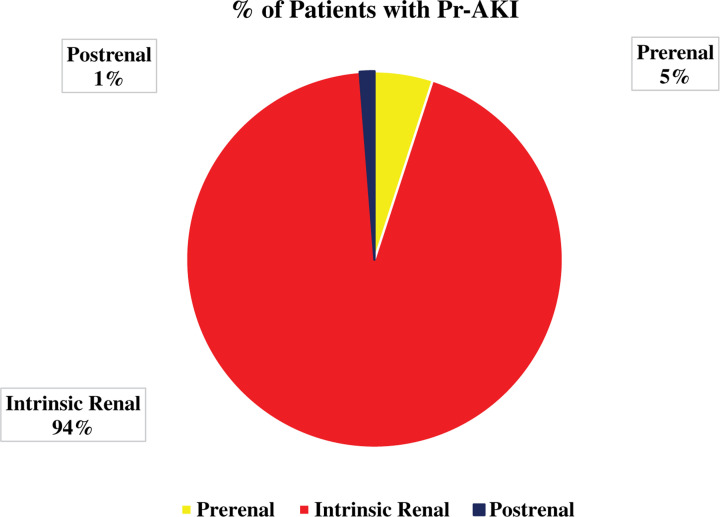
Distribution of cases of PRAKI according to category of acute kidney injury

Figure 3 showed that most cases of PRAKI fall into the intrinsic renal category (94%) as compared with prerenal (5%) and postrenal category (1%).

### [TIMP-2]*[IGFBP7] is a useful biomarker of PRAKI

**Figure 4:**
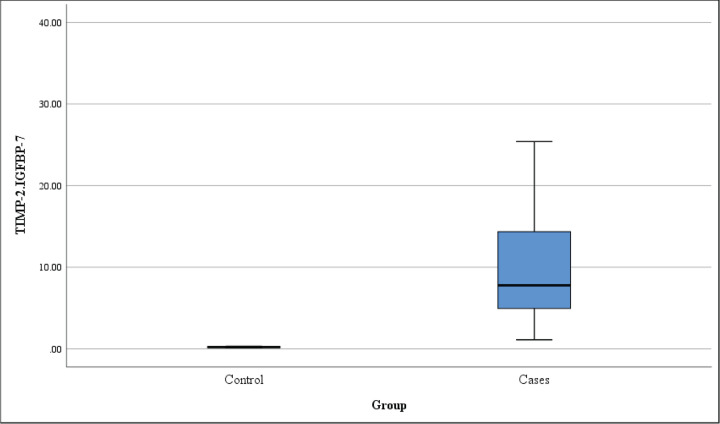
Box plot of TIMP7*IGFBP7 in PRAKI and control cases

Analysis of [TIMP-2]*[IGFBP7] as a diagnostic biomarker of PRAKI showed that [TIMP-2]*[IGFBP7] is statistically significant higher in PRAKI cases than controls (10 ± 7 vs. 0.2 ± 0.1; *P*<0.001) with the median is (8.0 vs. 0.2, respectively) as shown in Table 2 and Figure 4.

**Table 2:** Performance of urinary [TIMP-2]*[IGFBP7] for diagnosis of AKI

	Control	Cases	*P* value
	*N*=30	*N*=80	
TIMP-2*IGFBP-7			
Range	(0.1–0.3)	(1–33)	<0.001*
Mean ± SD	0.2 ± 0.1	10 ± 7	
Median	0.2	8	

IGFBP7, insulin-like growth factor-binding protein 7; TIMP-2, tissue inhibitor of metalloproteinases-2.

**Figure 5:**
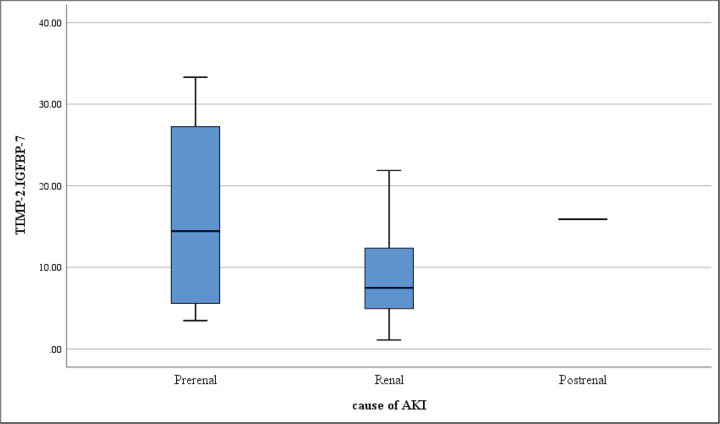
Box plot of the different categories of AKI among patients with PRAKI

Sub-analysis of PRAKI cases according to categories of AKI showed that the median [TIMP-2]*[IGFBP7] is higher in prerenal and postrenal causes as compared with intrinsic renal causes but without statistical significance (14.4(5.6–27.2), 15.9(15.9–15.9) and 7.5(4.9–12.5), respectively; *P*=0.303) as shown in Figure 5.

**Figure 6:**
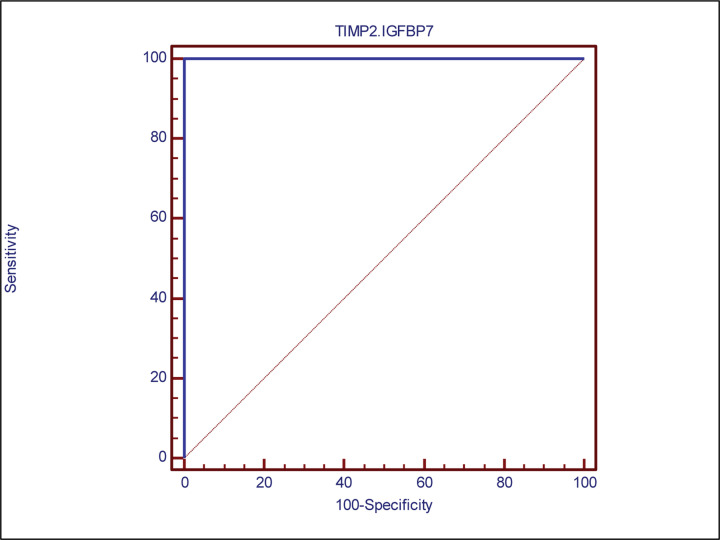
ROC curve of TIMP2*IGFBP7 as a diagnostic test for PRAKI AUC = 1.0 (95% confidence interval: 0.967–1) for the prediction of PRAKI; PRAKI, pregnancy-related acute kidney injury; AUC, area under the ROC curve.

ROC curve for prediction of PRAKI using [TIMP-2]*[IGFBP7] is shown in Figure 6. The AUC (95% CI) of [TIMP-2]*[IGFBP7] was 0.33 for prediction of PRAKI (0.967–1.0). Table 3 showed operating characteristics for [TIMP-2]*[IGFBP7] cutoff of 0.33. The sensitivity was 100% and the specificity was 100%. The NPV was 100% and the PPV was 100%**.**

**Table 3:** ROC curve analysis of [TIMP-2]*[IGFBP7] predicting AKI

Cutoff point	AUC	Sensitivity	Specificity	PPV	NPV	95% CI	*P* value
>0.33	1	100	100	100	100	0.967–1	<0.001*

## Discussion

AKI in pregnancy remains an important cause of morbidity and mortality in developing countries including Egypt. The definition, and therefore, the incidence of PRAKI varies widely in published literature, ranging from an increase in serum creatinine >0.8 mg/dl to the need of dialysis. The increase in serum creatinine may be a late event since it is decreased during normal pregnancy and may reach 0.6–0.7 mg/dl during the third trimester due to the combined effects of blood volume expansion, glomerular hyperfiltration, and decreased oncotic pressure as a consequence of hemodynamic and vascular changes that occur. Therefore, an increase of serum creatinine >0.8 mg/dl may reflect PRAKI after exclusion of prerenal causes [4]**.** AKIN and RIFLE criteria used to define AKI in the general population are not well validated in pregnancy because they depend on changes in serum creatinine [15].

The incidence of PRAKI in our study was 1.1% with the patient’s age ranged from 17 to 37 years with a mean of 26.1 ± 4.9 years. Most of the cases of PRAKI occur in third trimester and postpartum period (48 patients (60%) and 18 patients (23%) respectively).

The most common cause of PRAKI in our study was pre-eclampsia/eclampsia spectrum (61%), followed by sepsis (13.75%), and postpartum hemorrhage (11.25%). Our finding was in agreement with other previous studies in South Africa, Uruguay, Turkey, and Morocco that showed pre-eclampsia/eclampsia spectrum is the most common cause of PRAKI [16–19].

The characteristic histopathologic kidney lesion of pre-eclampsia/eclampsia is swelling and detachment of glomerular endothelial cells with subendothelial deposits in some cases that lead to capillary luminal obstruction. The altered hemodynamic abnormalities which are present in pre-eclampsia/eclampsia spectrum such as decreased renal plasma flow, 30–40% reduction in GFR, and renal vasoconstriction contribute to increased susceptibility to ischemic injury [20]. The other two common causes of PRAKI are sepsis and postpartum hemorrhage which lead to acute tubular necrosis. This makes the most common category of PRAKI is intrinsic AKI in 94% of cases with little contribution from pre-renal and postrenal categories.

We test the diagnostic performance of [TIMP-2]*[IGFBP7] in PRAKI. We found that [TIMP-2]*[IGFBP7] to be higher in cases of PRAKI as compared with controls, with higher levels noticed in prerenal and postrenal cases. We used a cutoff point of 0.33 ng/ml for the prediction of PRAKI with both sensitivity 100% and specificity 100% (95% confidence interval: 0.967–1.0, *P*<0.001). This is not in accordance to a previous study that recruited 66 pregnant women of them 44 developed PRAKI that failed to show any role of [TIMP-2]*[IGFBP7] in obstetric patients. This may be attributed to higher incidence of sepsis (33%) and shock (16%) in this critically ill small cohort with some patients had more than one co-morbidity [21].

Our study has some limitations. First, it is a single center study in small number of patients especially in the prerenal and postrenal categories of PRAKI which might affect the statistical power; but this is attributed to the low incidence of these categories in pregnancy. Second, in the intrinsic renal category of PRAKI, larger number of patients with different causes need to be recruited to investigate the possible role of [TIMP-2]*[IGFBP7] in the differentiation between them. Third, to complete the validation as a diagnostic marker, further research is needed to compare AKI patients with a larger number of patients who experience the same complications but without development of AKI.

## Conclusion

In conclusion, we report that PRAKI is still a common problem in developing countries with the third trimester and postpartum period are the most vulnerable period, pre-eclampsia/eclampsia spectrum is the most common cause of PRAKI, and most importantly [TIMP-2]*[IGFBP7] is a valuable tool in the diagnosis of PRAKI that needs further research on a large scale of patients to determine the ability to differentiate between different causes of PRAKI and to study its predictive value in the diagnosis of PRAKI through serial measurements in high risk pregnant patients.

## Abbreviations

ALT: alanine transaminase
AST: aspartate transaminase
CBC: complete blood cell count
CRP: C-reactive protein
IGFBP7: insulin-like growth factor-binding protein 7
IUFD: intrauterine fetal death
PRAKI: pregnancy-related acute kidney injury
TIMP-2: tissue inhibitor metalloproteinases-2
